# Facilitators and Barriers to Implementation of the EIT-4-BPSD Intervention

**DOI:** 10.1093/geroni/igab046.591

**Published:** 2021-12-17

**Authors:** Liza Behrens, Kiernan Riley, Marie Boltz, Ann Kolanowski, Kimberly Van Haitsma

**Affiliations:** 1 Ross and Carol Nese College of Nursing, Pennsylvania State University, University Park , Pennsylvania, United States; 2 Penn State University, College Station, Pennsylvania, United States; 3 Pennsylvania State University, University Park, Pennsylvania, United States; 4 Penn State, University Park, Pennsylvania, United States; 5 The Pennsylvania State University, University Park, Pennsylvania, United States

## Abstract

This study aimed to explore the perceptions of stakeholders (site champions, administrators, and front-line, social service, and activity staff) regarding the EIT-4-BPSD implementation strategy, including its utility, and the barriers and facilitators to implementation in real-world settings. A process evaluation included qualitative data from focus groups conducted with 93 stakeholders of 21 nursing homes (NHs) that implemented the EIT-4-BPSD strategy. Data were analyzed using a conventional content analysis. Emerging codes were sorted into categories then organized in meaningful clusters based on the domains of the RE-AIM framework. Challenges, facilitators, and contextual factors explain variability in implementation of EIT-4-BPSD strategy among NHs in six key categories: multi-stakeholder engagement, multi-level outcomes, process adaptations, uptake and utility of EIT resources, adoption barriers and facilitators, and future planning. Overall, stakeholders reported that the EIT-4-BPSD strategy can be successfully implemented in NHs and is helpful in improving staffs’ approach to BPSD.

